# Biomarkers of Remote Ischaemic Conditioning in Stroke and Cerebral Small Vessel Disease: A Narrative Review

**DOI:** 10.3390/neurosci7020040

**Published:** 2026-03-25

**Authors:** Marharyta Kamarova, Ali Alhashimi, Mudasar Aziz, Joyce Balami, Alison Buck, Madeline McGinnis, Arshad Majid, Ali Ali, Sheharyar Baig

**Affiliations:** 1 Sheffield Institute for Translational Neurosciences, Department of Neuroscience, University of Sheffield, Sheffield S10 2HQ, UK; rytakamarova@gmail.com (M.K.); mudasar.aziz@nhs.net (M.A.); joyce.balami@nhs.net (J.B.); arshad.majid@sheffield.ac.uk (A.M.); ali.ali@sheffield.ac.uk (A.A.); 2 University of Sheffield, Sheffield S10 2TN, UK; ali.alhashimi@nhs.net (A.A.); alison.buck@nhs.net (A.B.); m.mcginnis@sheffield.ac.uk (M.M.)

**Keywords:** remote ischaemic conditioning, stroke, cerebrovascular disease, biomarkers, neuroprotection, inflammation

## Abstract

Introduction: Remote ischaemic conditioning (RIC) is a promising treatment for neurological disorders. It involves cycles of temporary ischaemic stimulus, usually applied to a limb, and has shown significant improvement in neurological function in many trials. This review focuses on identifying and summarising the biomarkers of RIC that can enhance clinical practice and understanding of its mechanisms. Methods: A search was conducted in MEDLINE and EMBASE up to August 2025 using terms related to ischaemic conditioning. Studies were included if they were RCTs involving cerebrovascular disease, used RIC as treatment, and measured mechanistic biomarkers. We extracted and summarised data on study design, participant characteristics, RIC intervention protocols (including timing, frequency, duration, and pressure), biomarker types and measurement methods, timing of biomarker assessment, and main findings relating biomarker changes to clinical outcomes. Results: The review identified twenty-one RCTs examining biomarkers, including serum biomarkers, imaging markers, and other physiological indicators. Key biomarkers identified include systemic inflammatory cytokines and various imaging markers such as cerebral blood flow (CBF), white matter hyperintensities (WMH), and brachial artery flow-mediated dilation (BA-FMD). Conclusions: The evidence suggests that RIC modulates various biomarkers linked to neuroprotection and recovery. Reliable biomarkers of RIC would enhance the understanding of its mechanisms and improve targeted therapies. The clinical utility of these biomarkers requires further validation through large-scale trials. Standardised protocols and longitudinal studies are essential for optimising RIC therapy and improving patient outcomes in stroke and cerebral small vessel disease. Future research should focus on expanding our understanding of these biomarkers and their interactions with RIC, leading to more personalised and effective treatments.

## 1. Introduction

**Figure 1:**
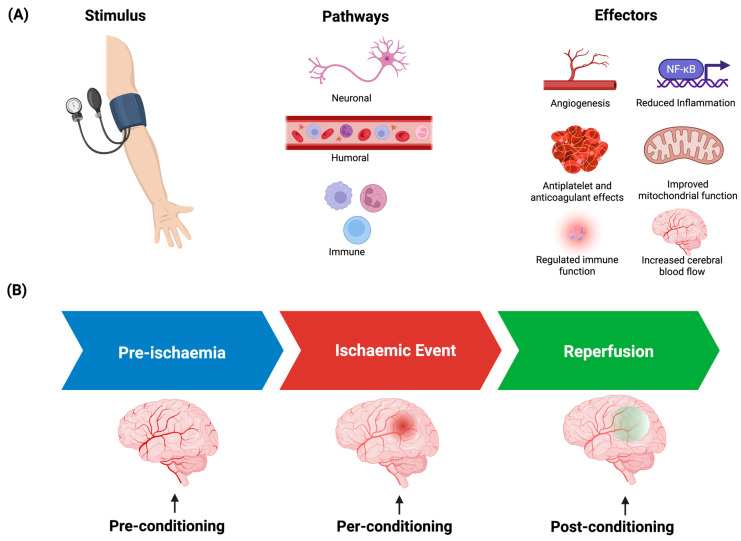
Potential mechanisms of action of RIC (**A**), and paradigms of treatment delivery (**B**).

Remote ischaemic conditioning (RIC) is a promising potential treatment in the management of cardiovascular and neurological disorders. RIC involves cycles of a temporary ischaemic stimulus, typically to a limb, applied via manual blood pressure cuff or automated device inflation. These cycles of ischaemia and reperfusion are thought to trigger a variety of innate physiological mechanisms that lead to end-organ effects such as alterations to tissue perfusion, immune function, and cellular bioenergetics (Figure 1A). Animal studies initially reported that these effects may mitigate tissue damage from experimental ischaemic events such as myocardial infarction and stroke [1]. Subsequently, there has been great interest in the use of RIC in neurological conditions, especially in stroke and cerebral small vessel disease (CSVD), as cerebral perfusion, inflammation, and impairments in cellular metabolism are commonly implicated aetiological factors in these disease states [2,3]. RIC can be administered before, during, or after an ischaemic event (known as pre-conditioning, per-conditioning, and post-conditioning, respectively; Figure 1B) in the context of stroke and is often delivered in a chronic manner in CSVD studies. While an overwhelming clinical benefit from large randomised controlled trials (RCTs) in these populations has not yet been observed, there have been promising signals suggesting that further exploration is warranted. Indeed, the mechanisms behind the effects of RIC are still not clearly understood, underlining the need for methods to evaluate how best to deliver the treatment or monitor its response. It is also becoming increasingly apparent that response to RIC varies between individuals, but a robust evidence base for predicting which patients will achieve physiological benefit from the intervention remains lacking.

Biomarkers represent measurable characteristics or substances that can either predict a course of disease or a response to treatment. The importance of studying biomarkers in RIC is fourfold. Firstly, there is a paucity of large RCTs that demonstrate clinical outcomes. Detecting changes that are plausibly linked to clinical outcomes in underpowered studies with short follow-up can provide justification for funding larger studies. Secondly, optimal dosing strategies for RIC are unclear. Undertaking studies with clinical outcomes often involves long durations of therapy and follow-up. Having biomarkers that change in a shorter space of time may allow identification of optimal dosing strategies (frequency, duration, pressure protocol) in a more efficient manner. Thirdly, studying biomarkers can help in the mechanistic understanding of RIC and may lead to the development of pharmacological targets for people with stroke or other cerebrovascular disorders. Fourthly, identification of early biomarkers of effect in RIC may provide the opportunity to assess treatment responses in individuals, thus identifying participant characteristics that may make the therapy more or less effective, moving therapy towards a more personalised approach to medicine.

**Figure 2:**
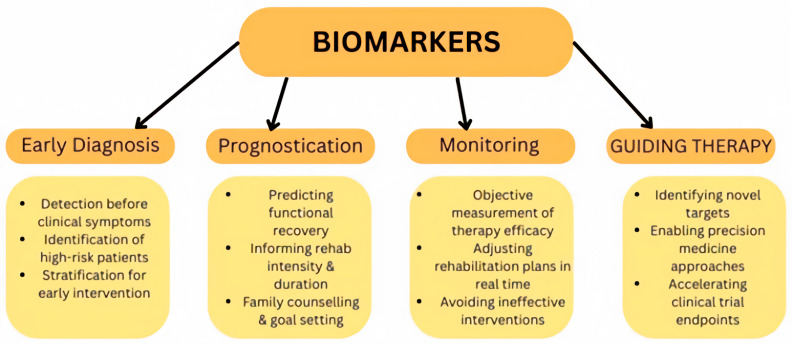
Potential benefits of biomarkers in remote ischaemic conditioning (RIC) research.

Literature on the established and frequently used biomarkers in RIC studies is lacking. This review represents the first systematic analysis of different types of biomarkers used in clinical RCTs of RIC in cerebrovascular disease. It is hoped that the findings of this review will help consolidate our understanding of how RIC exerts its effects and help future researchers in choosing appropriate biomarkers for their study design. Figure 2 highlights the key benefits of biomarkers in RIC research.

## 2. Methods

### Search Strategy and Study Selection

We conducted a search of MEDLINE via OVID (1946–August 2025) and EMBASE via OVID (1974–August 2025) on 1 August 2025. The search terms included “isch* conditioning,” “isch* preconditioning,” and “isch* postconditioning.” Additional studies were identified through reference lists, citation searches, and conference proceedings. Titles and abstracts identified from the search strategy were independently screened by three reviewers (SSB, MK, and AA1) and then updated and screened again by (MA, MK, AA1).

Studies were eligible for inclusion in the narrative review if they met the following criteria: were full-length articles, written in English, randomised controlled trials, involved participants with cerebrovascular disease (stroke, intracranial arterial stenoses, or cerebral small vessel disease), included RIC as the treatment, and had a biomarker as one of the outcome measures. Biomarkers were defined as physiological characteristics or biological substances that can be objectively evaluated to indicate pathological or biological processes or response to the intervention (RIC). Data extraction was performed independently by four authors (MA, MK, AA1, MM) using an identical data collection template. Information was extracted on methods, study participants, intervention, treatment duration, and primary and secondary outcomes. Biomarkers were categorised according to modality (serum, imaging, or other) and, within each modality, grouped by their primary biological function or mechanistic pathway (e.g., neuronal injury, inflammation, angiogenesis, neurotrophic factors, coagulation, lipids), with details recorded on the specific measurement methods and timing of assessment. The collected data were cross-checked among the authors, and any differences were resolved by referring to the original source material.

## 3. Results

**Figure 3:**
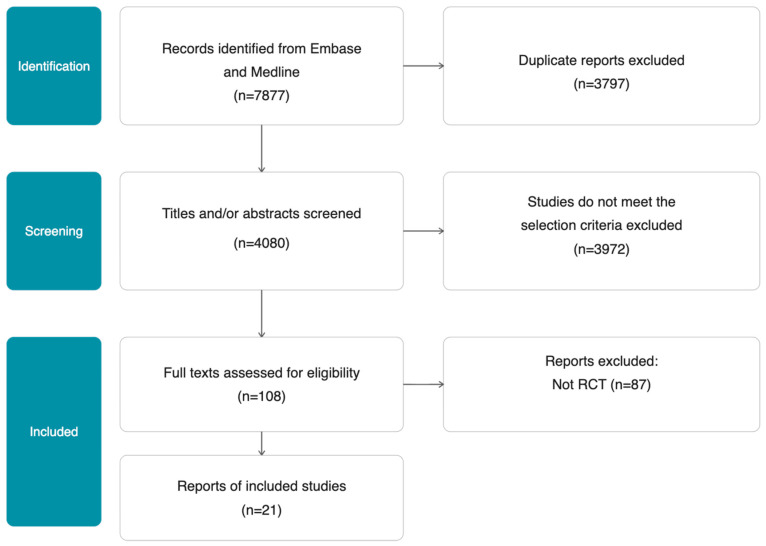
PRISMA flow diagram.

The initial search yielded 7877 records, of which 4080 remained after removal of duplicates. Following title and abstract screening, 3972 articles were excluded for not meeting the inclusion criteria, leaving 108 full-text articles assessed for eligibility. Of these, 21 randomised controlled trials met the inclusion criteria and were included in the review (Figure 3). The included studies investigated biomarkers across a range of modalities, which we categorised into three main groups: serum biomarkers (including markers of neuronal injury, inflammation, angiogenesis, neurotrophic factors, coagulation, and lipids), imaging biomarkers (including cerebral blood flow, white matter hyperintensity volume, and brachial artery flow-mediated dilation), and other biomarkers (including cerebrospinal fluid analytes, microRNAs, and optical coherence tomography angiography).

### 3.1. Serum Biomarkers

Eleven RCTs have examined serum biomarkers across acute ischaemic stroke [4,5,6,7,8,9], symptomatic intracranial carotid stenosis [10,11,12], and cerebral small vessel disease [13,14]. Serum biomarkers are relatively easy to sample, especially on a recurrent basis, and may therefore prove to be a low-cost method of detecting physiological effects of RIC. Hallmarks of effective serum biomarkers of RIC in stroke would include those that are strongly associated with stroke severity, have good test–retest reliability, have levels that are correlated with concentrations in the CSF, and are cost-effective.

### 3.2. Neuronal Injury

The most studied biomarkers of neuronal injury are S100B and neuron-specific enolase (NSE). These markers are known to rise in the hours to days after ischaemic injury [15,16], and are therefore of relevance in the aftermath of acute ischaemic stroke, although they have also been studied in more chronic settings of post-ischaemia [12].

S100B is a cytosolic protein found in glial cells. It is thought to be released upon neuronal cell death, peaking at 2.5 days and remaining elevated for up to 9 days in the plasma, and is linked to a pro-inflammatory state post-stroke [15,17]. Increased levels of S100B are associated with blood–brain barrier (BBB) breakdown and were found to be an independent risk factor for haemorrhagic transformation in thrombolysed stroke [18]. Clinically, S100B has been found to be correlated with infarct volume and functional outcomes such as ADL scores, whilst NSE, a neuronal cytosolic protein, is only correlated with infarct volume [15,16].

In acute ischaemic stroke, two studies found a decrease in serum S100B [5,8]. The RECAST2 trial found that S100B levels increased in the sham RIC group but not in the treatment group at day 4. S100B levels were found to be correlated with functional outcome (mRS at day 90) and baseline stroke severity. However, the first RECAST trial found no difference in S100B levels between groups at day 4, highlighting differences in RIC protocol as a possible factor [4].

An et al. conducted an RCT of 68 patients with AIS undergoing thrombolysis [8]. These patients received twice-daily RIC or standard medical therapy during inpatient admission and similarly demonstrated lower levels of S100B at discharge in the RIC group, which was associated with favourable neurological recovery (mRS scores 0–1 at 90 days). Participants in the RIC group had a significant increase in favourable recovery (71.9% vs. 50%). However, no formal correlation statistics were conducted. One point to consider in future studies is whether RIC has a greater effect on S100B levels in patients undergoing thrombolysis and/or mechanical thrombectomy.

### 3.3. Inflammation

The nature and timing of the inflammatory processes related to acute ischaemic stroke and cSVD are multifaceted, and it follows that the effects of RIC on these pathways will be similarly complex. During stroke, ischaemic tissue is thought to release reactive oxygen species and cytokines, as well as induce the expression of adhesion molecules, thus activating the vascular endothelium and causing infiltration of pro-inflammatory immune cells [19]. The further release of cytokines and chemicals such as MMP-9 by immune cells leads to cerebral oedema, blood–brain barrier disruption, and ultimately exacerbates neuronal cell death [20]. High levels of vascular inflammation also have pro-atherogenic effects [21], leading to increased risk of further stroke and affecting plaque stability [22].

RIC itself has been shown to have anti-inflammatory effects. A microarray study in healthy volunteers demonstrated that a single cycle of RIC suppresses expression of genes involved in inflammatory responses mediated by leukocytes, including downregulation of TLR4 and TNFα and upregulation of HSP70 [23].

Serum biomarkers of inflammation have been studied in AIS, sICAS, and cSVD. These include CRP, ICAM-1, IL-1, IL-6, IL-10, TNFα, TLR4, NF-κB, MMP-9, heat shock proteins, homocysteine, as well as levels of leukocytes. As seen in Supplementary Table S1, six RCTs found significant differences in at least one inflammatory biomarker in the treatment group compared to control after periods of RIC [4,6,7,10,12,13].

Beyond traditional plasma markers, cell count–derived indices have also been investigated as predictors of RIC response. In a secondary analysis of the RICAMIS trial, Cui et al. (2025) divided patients based on their platelet-to-neutrophil ratio (PNR) during admission. They found that RIC was associated with a 90-day excellent functional outcome in the low PNR group (<30.98; 60.9% vs. 50.3%, adjusted RD 11.3%), but not in the high PNR group. The pattern suggests that PNR may identify patients with a more inflammatory and prothrombotic profile, and it is precisely this subgroup that appears to gain the most from RIC therapy [24].

### 3.4. Matrix Metalloproteinases (MMPs)

Matrix metalloproteinases, especially MMP-9, play a central role in breaking down the extracellular matrix and spike dramatically after ischaemic stroke. Higher MMP-9 levels track closely with infarct size, stroke severity, functional outcomes, and blood–brain barrier disruption, particularly when oedema is present [13,25,26]. It is also a useful predictor of haemorrhagic transformation in patients who receive thrombolysis for acute ischaemic stroke.

Interestingly, An et al. conducted an RCT and found no meaningful differences in MMP-9 levels at discharge between thrombolysed patients who received remote ischaemic conditioning (RIC) and those who did not [8]. Both groups had one case of intracerebral haemorrhage, but it is worth noting that MMP-9 levels might tell a different story earlier on, since the risk of bleeding peaks within the first 36 h [27]. This raises the possibility of combining RIC with drugs that specifically target MMP-9 to further lower ICH risk [28]. What makes MMP-9 particularly valuable as a biomarker is that it responds to inflammatory signals throughout the body, unlike MMP-2 and MMP-14, which are always active [29]. Xu et al. showed that 6 months of daily RIC led to a significant drop in MMP-9 in patients with symptomatic intracranial atherosclerosis [12]. However, the RECAST and RECAST2 trials did not find significant differences between RIC and control groups in MMP-9 levels, though there was a non-significant uptick in the sham group on day 4 [4,5]. These mixed results likely reflect differences in patient populations and the duration of RIC administration.

### 3.5. Cytokines

If neurons become ischaemic, they trigger the release of both pro- and anti-inflammatory cytokines. Clinical outcomes often hinge on the balance between these competing signals; low levels of the anti-inflammatory cytokine IL-10, for instance, are linked to early neurological decline [30]. IL-10 deficiency also goes hand-in-hand with elevated TNFα, MMP-9, and other inflammatory markers, leading to larger infarcts in animal studies. Doll et al. reviewed how cytokines change after stroke and found that IL-1β rises in cerebrospinal fluid by days 2–3, while IL-6 increases in both CSF and blood [31]. The pattern for TNFα is less clear-cut.

In an important study, Meng and colleagues showed that 30 days of RIC in elderly patients with symptomatic intracranial atherosclerosis led to significant drops in C-reactive protein, IL-6, and white blood cell counts, changes that correlated with fewer strokes and TIAs [10]. Since IL-6 is a key player in inflammation and activates the vascular lining [32], this suggests RIC might influence several inflammatory pathways at once. However, RECAST found no significant IL-6 changes after 4 days of RIC in acute stroke patients [4], which could be due to differences in how RIC was delivered or fundamental differences between chronic and acute inflammatory responses. RECAST also showed a brief dip in TNFα after RIC that did not last beyond day 4, suggesting the immunomodulatory effects might be short-lived [4].

Vascular inflammation is central in cerebral small vessel disease, though it is still not clear whether it is a cause or consequence. The working theory is that risk factors like high blood pressure and elevated blood sugar set off inflammatory responses in blood vessels [33]. RIC has had modest success at best in affecting inflammatory markers in small vessel disease. Liao et al. found no significant differences in CRP, IL-6, or TNFα between RIC-treated patients and controls, even though cognitive function improved [14]. However, Wang et al. demonstrated a significant reduction in homocysteine, a pro-inflammatory marker, along with improvements in white matter hyperintensity volume and cognitive abilities after a year of RIC in small vessel disease patients [13]. The differences can be attributed to variations in patient characteristics and baseline cognitive status.

### 3.6. Integrins

Intercellular Adhesion Molecule 1 (ICAM-1) plays a vital role in inflammatory cell recruitment through the vascular endothelium, including through the blood–brain barrier [26,27]. Its levels have been shown to rise following periods of cerebral ischaemia [34]. Feng et al. [6] compared 6 months of daily RIC to standard medical treatment in patients with AIS, measuring levels of ICAM-1 and the vasoconstrictor endothelin-1, as well as cognition, ADL scores, and cerebral blood flow. The RIC group showed significantly lower levels of ICAM-1 and endothelin-1 after 6 months compared to the control group, and both biomarkers showed a positive correlation with pulsatility indices of the MCA and brachial artery. The authors speculate that the improvements in cerebral perfusion (as measured by MCA mean blood flow velocity) and, therefore, reduction in ischaemia may have reduced levels of these biomarkers in the RIC group. However, the effect of RIC on ICAM-1 levels in the short term remains unclear, and whether a reduction in ICAM-1 levels would contribute to an immediate window of protection with RIC.

### 3.7. Transcription Factors

NFκB is a vital transcription factor involved in inflammatory responses and leads to gene expression of cytokines [35]. It can be activated through numerous pathways, including TLR4. Ji et al. [7] compared levels of NFκB and TLR4 after 8 weeks of RIC or routine medical care in patients with AIS. The authors found that the RIC group had lower levels of these inflammatory substances compared to the control group. TLR4 levels were inversely correlated with measures of collateral circulation (rLMC score). The downstream effects of this pathway (such as interleukins and cell adhesion molecules) were not sampled, which may provide greater insight into the possible effects of RIC on this pathway.

### 3.8. Heat Shock Proteins

Heat shock proteins (HSPs) are protective proteins that cells express under stress conditions. Their protective mechanisms include scavenging reactive oxygen species and inhibiting apoptotic pathways. Studies in experimental stroke models have demonstrated benefits from HSP27 [36]. Clinical data show that HSP27 levels in stroke patients increase rapidly following the acute event, reaching maximum concentrations between 24 and 48 h post-stroke, then declining to baseline by day 30 [37].

Two RIC trials investigated HSP27, with RECAST showing an increase in both total and phosphorylated HSP27 after 4 days of RIC [4]. NIH Stroke Scale (NIHSS) scores were significantly lower in the RIC group at 90 days. No differences were found in other HSPs. An et al. reported no significant difference in HSP27 between RIC and controls at discharge, although they did not measure phosphorylated HSP27 [8]. The RECAST2 trial could not present HSP27 data due to unreliable assays [5]. Given the longer RIC protocol in An et al.’s study, further research is needed to clarify the relationship between RIC, HSP27, and thrombolysis [5].

### 3.9. Angiogenesis

Angiogenic factors, particularly vascular endothelial growth factor (VEGF) and basic fibroblast growth factor (bFGF), support stroke recovery through their effects on vascular and neuronal growth [38]. These angiogenic biomarkers are useful in trials testing interventions that aim to improve collateral blood flow and reduce recurrent stroke risk.

Preliminary results from the EPIC-SICAS trial showed elevated bFGF and VEGF levels after 10 days of RIC, raising the possibility that the treatment promotes angiogenesis and neuroprotection [11]. An et al. also found increased VEGF, but not bFGF, at discharge in AIS patients treated with RIC [8].

Both studies employed similar RIC protocols, though EPIC-SICAS lasted 10 days and An et al. lasted for the duration of hospital stay (average 11.2 days) [8]. Despite these similarities, disparities in patient populations (sICAS vs. AIS) and sample size may account for the different results.

Yu et al. (2023) examined whether RIC could be used to improve motor recovery after stroke by promoting angiogenesis [9]. They found that patients who received RIC showed better lower limb function as assessed using the Fugl–Meyer Assessment, and this improvement correlated with higher epidermal growth factor (EGF) levels. They also found that other angiogenic factors such as PDGF and VEGF remained unchanged. The correlation between EGF and motor recovery raises the possibility that EGF could serve as a useful biomarker for tracking rehabilitation progress. That said, these findings need replication in larger studies before firm conclusions can be drawn about RIC’s role in post-stroke recovery.

### 3.10. Neurotrophic Factors

Brain-derived neurotrophic factor (BDNF) is a growth factor that has been associated with stroke recovery through its effects on neuronal survival, promoting neuroplasticity and reducing inflammatory cascades that lead to neuronal apoptosis [39]. BDNF therapy has also been shown to reduce infarct size in animal models of stroke [40]. Promoting neurotrophic effects thus has possible applications in both the acute and chronic phases of ischaemic stroke.

Two studies have investigated the neurotrophic agent BDNF in RIC. An et al. investigated patients with acute ischaemic stroke undergoing thrombolysis, and Xu et al. investigated patients with sICAS [8,12]. The latter study noted increased serum BDNF levels after 6 months of daily RIC in the treatment group, whilst the former found no difference in BDNF levels at discharge between the two groups. As with previous biomarkers, the differences here may be explained by the chronicity of RIC treatment, the time point at which BDNF is measured, and the patient pathophysiology in question. It is crucial to consider that serum BDNF levels may not fully reflect levels within the central nervous system; this distinction suggests that peripheral BDNF measurements might not provide a complete picture of the neurotrophic processes occurring in the brain.

### 3.11. Coagulation and Platelet Function

Markers of coagulation, endogenous anticoagulant pathways, and platelet function are likely to be of particular importance when measuring the effect of RIC on recurrent stroke. Results from RIC in trials of cardiovascular disease have shown some early effect on platelet function. For example, the ERIC-PPCI trial examined the effect of RIC on the thrombotic status of patients with STEMI undergoing PPCI, with patients also receiving dual antiplatelet therapy (DAPT) as standard [41]. Although the RIC group showed a longer time to in vitro thrombotic occlusion than the sham group at discharge, this effect was not maintained at 8 weeks, nor were there between-group differences in other measures of thrombosis (such as clot formation and fibrinolysis) throughout the trial.

Two studies on cerebrovascular disease have looked at how RIC affects coagulation biomarkers, working with patients who had sICAS and cSVD [10,13]. In one study, Meng et al. studied patients with symptomatic intracranial arterial stenosis and found that 30 days of daily RIC led to significant drops in coagulation markers compared to the sham treatment group [9]. In particular, this study detected an increase in the fibrinolytic agent tPA, and reductions in fibrinogen, PAI-1, and platelet aggregation rates, all of which contribute to haemostasis. Participants received either clopidogrel 75 mg as monotherapy (88%), or as dual antiplatelet therapy with aspirin 100 mg (12%). Wang et al. found no difference in fibrinogen levels between long-term RIC and control in patients with cSVD. Whilst the breakdown of antiplatelet medications used by patients was not given, the authors commented that this was controlled for similarly between the RIC and sham groups.

A novel global biomarker of coagulation, the fractal dimension (df) of clot microstructure, has recently been applied in stroke research. Derived from viscoelastic rheometry of whole blood, df reflects the density and complexity of fibrin architecture: higher values indicate tighter, more thrombogenic clots that are resistant to fibrinolysis. In a 2025 crossover trial of patients with lacunar stroke, Norregaard et al. found that acute ischaemic preconditioning actually increased df, even though patients were on antiplatelet therapy at the same time [42]. This suggests that conditioning stimuli might temporarily enhance thrombogenic potential. Incorporating df alongside conventional plasma markers may therefore provide a more integrated picture of coagulation dynamics.

Although widely used in clinical practice, D-dimer has been little explored in the context of remote conditioning. Norregaard et al. (2025) [42] did not find any significant changes in D-dimer levels among lacunar stroke patients after ischaemic preconditioning. This suggests that D-dimer is not particularly sensitive for detecting vascular changes triggered by conditioning.

It is apparent that there is no clear picture as to how RIC might influence thrombosis. What the evidence suggests is that whether patients are taking one or a combination of antiplatelet medications, this has to be factored in when examining how RIC affects thrombosis markers.

### 3.12. Lipids

Lipids and cholesterol are well-known risk factors for cSVD and stroke [43]. Oxidised LDL has been shown to be particularly atherogenic [44] and is linked to poor functional outcome in stroke [13].

In cSVD, Wang et al. compared lipid profiles at baseline and 1 year for the control and treatment groups of cSVD patients receiving once-daily RIC or sham treatment. Compared to the control, the treatment group showed significant reductions in triglycerides, cholesterol, and LDL at follow-up. There was a significant difference in the primary outcome of WMH volume, as well as improved visuospatial abilities on cognitive testing and reduced pulsatility indices of the MCA. The authors did not measure levels of oxidised LDL, which may be another biomarker to consider, given its atherogenic properties. Clearly, these markers are known risk factors for stroke, but it remains unclear the mechanism by which RIC can influence them, and there is a lack of pre-clinical studies investigating this.

More recently, lipoprotein(a) [Lp(a)] has emerged as a clinically relevant biomarker in the context of remote ischaemic conditioning. In a post hoc analysis of the RICA trial, Wu et al. (2025) found that high Lp(a) levels were linked to a greater risk of recurrent ischaemic stroke in patients with symptomatic intracranial atherosclerotic stenosis [45]. Each doubling of Lp(a) increased recurrence risk by ~20% (HR 1.20, 95% CI 1.10–1.30), and patients with levels above 17.4 mg/dL had significantly greater risk than those below this threshold. Importantly, the protective effect of RIC appeared strongest in patients with elevated Lp(a): recurrence occurred in 16.7% of the RIC group compared with 22.6% of controls (adjusted HR 0.67, 95% CI 0.47–0.96). However, RIC did not change Lp(a) levels over time, which suggests Lp(a) works better as a way to stratify patients rather than as a target that can be modified with conditioning. This suggests that Lp(a) could help determine which patients are most likely to benefit from RIC. With Lp(a)-lowering therapies now making their way into clinical practice, there might be real value in pairing these drugs with conditioning strategies for secondary prevention in high-risk ICAS patients.

### 3.13. Imaging

#### 3.13.1. Cerebral Blood Flow

Perfusion-weighted imaging is a reliable way to assess cerebral ischaemia through functional imaging. It measures key parameters such as cerebral blood flow (CBF), cerebral blood volume (CBV), and mean transit time (MTT), all of which can help predict outcomes and evaluate how well treatments are working [46]. CBF is especially useful for identifying the infarct core and determining whether penumbral tissue can still be saved [47]. Li et al. (2018) studied how remote ischaemic conditioning (RIC) affects CBF [48]. They measured CBF and MTT before treatment, at day 14, and at 90 days. At 14 days, there were no meaningful differences between the control and intervention groups. But by day 90, the intervention group showed a notable decrease in MTT and an increase in CBF compared to controls. The underlying mechanisms of the RIC-mediated enhancement of CBF are still under investigation. Animal studies hint that it may work through nitric oxide (NO) pathways; experiments in mice showed that NO helps maintain blood flow in the liver’s small vessels [49]. Another study suggested that RIC might prevent collateral blood vessels from collapsing during ischaemic strokes, which would improve CBF [50].

Xu et al. (2018) further investigated the influence of RIC on blood flow velocity through several intracranial vessels [12]. Since we know intracranial blood flow velocity correlates with overall CBF [51], they used transcranial Doppler ultrasonography to measure mean blood velocities in the middle cerebral, anterior cerebral, posterior cerebral, vertebral, and basilar arteries, both before treatment and at 6 months. Their results showed that the RIC group had significantly better blood flow through all these cerebral arteries at 6 months, both compared to their own baseline and to the control group.

#### 3.13.2. MRI for White Matter Disease

White matter hyperintensities of presumed vascular origin (WMHs) are lesions secondary to cerebral microangiopathy, identifiable as hyperintense lesions on T2-weighted or FLAIR MRI scans. These lesions are recognised as indicators of cerebral small vessel disease (cSVD), as well as risk factors for future ischaemic stroke [52]. They also serve as viable outcome measures in assessing the therapeutic efficacy of novel treatments targeting cSVD [53]. The pathophysiology of the development of WMHs is not fully elucidated, but two initial theories have been proposed. The first posits that WMHs arise from chronic hypoperfusion, with imaging studies demonstrating their localisation in areas of reduced cerebral blood flow (CBF) [54]. The second theory points to endothelial dysfunction in cerebral vessels, which affects the blood–brain barrier and capillary bed and makes vessels more permeable. Studies showing contrast accumulation in WMHs support this idea [55]. More recently, researchers have started combining these theories, proposing that both mechanisms work together to cause WMHs and, in turn, cSVD [56].

Two clinical trials have investigated the impact of remote ischaemic conditioning (RIC) on WMH progression in cSVD [13,57]. Mi et al. (2016) examined the effect of long-term RIC therapy on cSVD. Their study showed a significant decrease in WMH volume after one year of RIC treatment, from 6.06 cm^3^ (4.67–10.95) before treatment to 4.19 cm^3^ (2.96–7.25), while the control group saw an increase in WMH volume [57]. Wang et al. (2017) corroborated these findings, showing significant reductions in WMH volume with a similar prolonged RIC protocol to Mi et al., which consisted of twice-daily RIC for one year [13]. Their results indicated a notable reduction in the intervention group’s WMH volume compared to pre-intervention and control group measurements. RIC is thought to improve WMH volume by enhancing CBF, which occurs through promoting angiogenesis, collateral formation, and vascular remodelling [58].

### 3.14. Endothelial Markers

Brachial artery flow-mediated dilation (BA-FMD) is recognised as a reliable marker of endothelial dysfunction and has been validated for assessing the risk and prognosis of cardiovascular events [59]. The endothelium is essential for maintaining vascular homeostasis; it regulates vascular tone, releases vasodilators, and controls coagulation by mediating platelet aggregation [60]. When endothelial function becomes impaired, it contributes to atherosclerosis development through abnormal leukocyte adhesion, smooth muscle proliferation, and thrombosis, which raises the risk of cardiovascular diseases [60]. Recent studies have demonstrated that patients who have suffered a stroke exhibit reduced BA-FMD, which is associated with a poorer prognosis. This aligns with their heightened risk of developing cardiovascular diseases [61].

In an RCT conducted by Hyngstrom et al. (2020), 24 chronic stroke patients participated. The intervention group received RIC on their lower limb every two days for two weeks. BA-FMD measurements were taken before the intervention and 24–48 h after the last RIC session. This group exhibited a notable improvement in FMD percentage, from 5.4 ± 4.7% pre-intervention to 7.8 ± 4.4% post-intervention, whereas the sham group showed no significant change in FMD (3.5 ± 3.9% pre-intervention to 2.4 ± 3.1% post-intervention) [62]. Of note, the protocol utilised highlights that the improvements in endothelial function are systemic, as FMD measurements were taken from the upper limbs rather than the lower limbs where RIC was applied. Another study by Kate et al. (2019) compared the effects of 4 versus 6 cycles of RIC therapy over 12 weeks on BA-FMD. They found no significant differences in BA-FMD either on day 7 or day 90 of the trial [63]. It is not known whether the effects of RIC delivered at the lower limb (with a larger muscle volume and potentially greater ischaemic signal) exert a greater effect than upper limb RIC.

More recently, Norregaard et al. (2025) assessed endothelial function using brachial FMD in patients with recent lacunar stroke [42]. While baseline arterial diameter increased following a two-week protocol of ischaemic preconditioning, there was no improvement in FMD percentage compared with sham treatment. This difference from earlier studies points to potential disease-specific variability and suggests that BA-FMD may respond less to RIC when small vessel pathology is already established.

The underlying biological mechanisms for the improvement of BA-FMD following RIC are not yet fully understood. However, several pathways have been implicated. Research indicates that the reduction in nitric oxide availability negates the improvement in endothelial function and BA-FMD observed with RIC [64]. Another study found that blocking potassium ATP channels prevents RIC’s vasoprotective effects, so there was no improvement in BA-FMD compared to controls [65]. How these pathways connect and regulate each other is not yet clear.

### 3.15. Discussion

Our review highlights two broad classes of biomarkers in remote ischaemic conditioning (RIC) research: those reflecting reduced neurological injury and those representing recovery or neuroplasticity. Injury-associated biomarkers such as S100B, NSE, and inflammatory mediators (IL-6, TNFα, ICAM-1, TLR4/NF-κB, MMP-9) change rapidly after acute ischaemic stroke (AIS) and may capture early treatment effects. S100B specifically correlated with baseline stroke severity and 90-day mRS in RECAST/RECAST2. Some trials found lower S100B levels after RIC, though results varied depending on the protocol used. Several studies also found that RIC affected inflammatory and matrix-remodelling pathways, but these effects depended heavily on timing and the patient population being studied.

Markers of recovery and neuroplasticity including angiogenic factors (VEGF, bFGF, EGF), neurotrophic factors (BDNF), endothelial function (BA-FMD), and perfusion metrics (CBF/MTT), were more closely associated with subacute and chronic phases of disease, supporting a role in longer-term repair and vascular remodelling. Increases in EGF were linked to motor recovery, BA-FMD improved after short courses of RIC, and imaging at 90 days showed improvements in CBF and MTT.

When considering disease context, AIS is best served by fast-responding serum biomarkers of injury and inflammation, short-term vascular measures such as BA-FMD, and perfusion imaging to capture delayed haemodynamic benefits. In contrast, cerebral small vessel disease (cSVD) lends itself to long-horizon imaging endpoints such as white matter hyperintensity (WMH) volume and vascular risk markers (homocysteine, lipid profile), with transcranial Doppler indices providing additional functional readouts. Notably, inflammatory modulation is less consistent in cSVD than in AIS, and the effect of RIC may require longer exposure to become apparent.

The biomarkers most consistently linked to clinical outcomes were S100B (associated with mRS and severity in AIS), homocysteine and lipid improvements (tied to WMH volume and cognition in cSVD), ICAM-1/endothelin-1 (linked to cerebral haemodynamic status), EGF (associated with motor recovery), and CBF/MTT (showing perfusion improvements). MMP-9, while biologically linked to severity and haemorrhagic transformation, showed inconsistent trial-level modulation, underscoring the importance of sampling windows.

From a practical perspective, serum biomarkers are cheap and easy to measure repeatedly, but they are prone to variability from issues such as processing delays, storage conditions, daily fluctuations, and assay performance issues (like the HSP27 assay problems seen in RECAST2). CSF gives a better image of what is happening in the CNS, but it is invasive and not practical for repeated measurements. Imaging biomarkers offer mechanistic and prognostic insight but are costlier, require standardised protocols, and may be less available. BA-FMD is feasible but operator-dependent. Co-interventions such as antiplatelet therapy can confound coagulation biomarker interpretation. Selection of biomarkers should therefore consider timing, biological half-life, test–retest reliability, and context of use.

Biomarkers in RIC research can be grouped by function: diagnostic markers reflecting acute neuronal injury (e.g., S100B, NSE); prognostic markers correlating with infarct volume or functional outcome (e.g., MMP-9, homocysteine, Lp(a)); pharmacodynamic markers capturing RIC’s biological effects such as inflammatory modulation, angiogenesis, or cerebral perfusion; and predictive markers identifying subgroups likely to benefit from RIC. Distinguishing these roles is essential for appropriate biomarker selection and trial design.

In acute ischaemic stroke (AIS), pair fast-responding serum injury and inflammation markers with pragmatic vascular measures and subacute perfusion imaging to capture early damage and evolving haemodynamics. In cerebral small vessel disease (cSVD), prioritise longitudinal imaging, particularly white matter hyperintensities (WMH), together with vascular risk biomarkers under prolonged remote ischaemic conditioning (RIC). Select biomarkers to match an explicit mechanistic hypothesis (injury containment vs. recovery promotion) and pre-specify sampling time points; standardise sample handling and report assay reliability. Where appropriate, use composite panels spanning injury and recovery domains to strengthen trial design and inform dosing strategies.

### 3.16. Other Potential Biomarkers of RIC

Remote ischaemic conditioning (RIC) shows promise as a treatment for stroke and cerebral small vessel disease (cSVD). Finding biomarkers linked to RIC could help improve its efficacy and clarify the mechanisms behind it. Research in cardiology and animal models has highlighted kallistatin and stromal-derived factor 1α (SDF-1α) as potentially important players in RIC’s neuroprotective effects. Kallistatin levels rise after RIC and help protect blood vessels from damage through several mechanisms [65,66,67]. SDF-1α supports tissue regeneration by boosting stem cell activity and has been found at higher levels in stroke models [68,69,70]. Inflammatory biomarkers have been well studied in stroke and cSVD, as inflammation plays a key role in these conditions [20,21,22,23]. RIC appears to protect the brain by modulating inflammation [24]. Two important inflammatory markers in this context are ICAM-3 and P-selectin, which have been linked to adverse stroke outcomes, with experimental evidence suggesting that preconditioning-mediated suppression of tumour necrosis factor (TNF) reduces P-selectin upregulation and downstream leukocyte adhesion, providing a plausible mechanistic pathway for these associations [71,72,73,74,75,76,77,78,79]. Glucagon-like peptide-1 (GLP-1) analogues show neuroprotective properties in animal models and may play a role in RIC-mediated neuroprotection by increasing cerebral blood flow [80,81,82,83]. DNA methylation could be another useful biomarker, as RIC triggered widespread changes in DNA expression and methylation in patients with a SAH [84,85,86]. MicroRNAs (miRNAs) that change with RIC in animal studies look promising as stroke biomarkers [87,88,89,90,91,92]. Optical coherence tomography angiography (OCTA) is an emerging non-invasive technique for assessing retinal blood flow, which reflects what is happening in cerebral small vessels [93,94,95,96]. Cerebrospinal fluid (CSF) biomarkers such as S100B, TNF-α, IL-6, neurofilament light, and matrix metalloproteinases have been linked to stroke severity and white matter degeneration in cSVD [97,98]. Recent work has explored haemorheological biomarkers as mechanistic targets of RIC. Blauenfeldt et al. (2025) found that RIC significantly reduced red blood cell aggregation, pointing to erythrocyte aggregation as a potentially useful new biomarker for RIC’s effects in acute stroke [99]. Using artificial intelligence alongside biomarker data and imaging could change how we predict and manage stroke and cSVD.

## 4. Conclusions

To determine if there is potential for a biomarker to measure the physiological effects of remote ischaemic conditioning (RIC) in cerebrovascular disease, there is a need to establish an association between ischaemic stroke and changes in biomarker levels. Important factors to therefore consider are when biomarker changes happen, how stable they are in serum, and whether they are linked to clinical outcomes like stroke severity or recurrence. Biomarkers should accurately reflect the cerebrospinal fluid (CSF) environment and be economical to test [100]. However, lumbar puncture is invasive, limits feasibility in acute stroke, and is impractical for serial monitoring in RIC trials. For this reason, serum biomarkers remain more clinically scalable.

Implementing novel biomarkers for RIC assessment in stroke and cSVD faces challenges. Trying to determine whether RIC actually influences biomarker change is difficult due to the complex nature of these conditions. There is a great need for longitudinal studies to understand how biomarkers change over time and how they relate to clinical outcomes, but these studies require significant resources. In addition, stroke pathology and biomarker responses can vary across different ethnic groups, so there is a need for studies that include diverse populations.

The persistence of biomarker changes post-RIC is crucial for determining the timing and frequency of RIC interventions. There is a need to explore inflammatory biomarkers such as high-sensitivity C-reactive protein (hsCRP) and see how they affect long-term recovery. Cost and accessibility are also significant issues. There is a need to balance using inexpensive, widely available markers like hsCRP against more specific but expensive options such as cerebral blood flow imaging.

Preclinical models offer valuable mechanistic grounding for understanding the variability seen in human RIC trials. In rodent MCAO paradigms, remote ischaemic conditioning reliably enhances neuroplasticity, including BDNF upregulation [101], preserves blood–brain barrier integrity by attenuating MMP-9 signalling and limiting tight junction degradation, and shapes inflammatory responses within defined temporal windows. Crucially, these effects unfold during controlled early reperfusion periods spanning hours to days, which allows biomarker changes to be more confidently attributed to the conditioning stimulus itself [102].

The picture in clinical studies is more complex. Cytokine and MMP-9 responses vary substantially across trials, and this heterogeneity is unsurprising given the range of conditioning paradigms employed, pre-, peri-, or post-stroke, alongside differences in sampling timing, stroke chronicity, and co-interventions including thrombolysis and antiplatelet therapy. This divergence from animal data therefore speaks more to translational complexity than to any fundamental mechanistic inconsistency. What it does highlight, however, is the importance of pre-specified sampling windows and biomarker selection grounded in the temporal dynamics established through experimental work; without this, the signal is too difficult to interpret.

### 4.1. Limitations

This review has several limitations. Firstly, the variability in RIC protocols across the included studies makes it hard to draw firm conclusions about how well specific biomarkers perform. Many of the trials also had relatively small sample sizes, which limits how much the findings can be generalised. In addition, many studies did not account for potential confounding factors such as differences in patient demographics, comorbidities, and other treatments patients were receiving. The review only included trials published in English, which may introduce language bias and miss relevant studies published in other languages.

Further large-scale, standardised, and longitudinal studies are needed to validate these findings and establish robust biomarkers for RIC in cerebrovascular diseases.

### 4.2. Conclusions

This review synthesises a wide array of evidence from various serum, imaging, and functional biomarker studies in the context of RIC in cerebrovascular diseases. The ability of RIC to modulate biomarkers and affect physiological responses downstream opens avenues for more targeted and individualised therapy in stroke and cSVD. Further exploration of biomarkers is crucial not only for elucidating the mechanisms of RIC but also to move towards a more personalised approach to medical therapy with RIC, potentially improving clinical outcomes for some patient groups and avoiding futile and burdensome treatments for others. RIC engages with multiple measurable physiological and biochemical pathways. Future work is needed to quantify these effects in a systematic way and use these measures as surrogate end points.

## Data Availability

No new data were created or analyzed in this study. Data sharing is not applicable to this article.

## Supplementary Materials

The following supporting information can be downloaded at: https://www.mdpi.com/article/10.3390/neurosci7020040/s1, Table S1: Summary of Biomarkers Investigated in Remote Ischemic Conditioning for Cerebrovascular Disease.
