# Supplementary material for: Biomarkers of Remote Ischaemic Conditioning in Stroke and Cerebral Small Vessel Disease: A Narrative Review
Source: NeuroSci. 2026 Mar 25;7(2):40. doi: 10.3390/neurosci7020040 (PMC13118872; doi:10.3390/neurosci7020040)
Supplement: Supplementary file 1 [file neurosci-07-00040-s001.zip › neurosci-4138007-supplementary.pdf]

Table S1. Summary of Biomarkers Investigated in Remote Ischemic Conditioning for Cerebrovascular Disease.

Biomarker Category	Specific Biomarker	Sample Type	Disease Context	Effect of RIC	Key Clinical Correlation
**NEURONAL INJURY MARKERS**	S100B	Serum	AIS, sICAS	↓	Correlates with stroke severity and 90-day mRS; reduced in RIC groups
	NSE	Serum	AIS	↔	Correlates with infarct volume; inconsistent RIC effects
**INFLAMMATORY CYTOKINES**	IL-6	Serum/CSF	AIS, sICAS, cSVD	↓ in sICAS/cSVD	Reduced after prolonged RIC; no change in acute stroke
	IL-10	Serum/CSF	AIS	N/A	Low levels linked to poor outcomes
	TNFα	Serum/CSF	AIS, cSVD	Brief ↓	Short-lived reduction; variable in chronic disease
	IL-1β	Serum/CSF	AIS	N/A	Rises in CSF days 2-3 post-stroke
	CRP	Serum	sICAS, cSVD	↓	Reduced after 30 days RIC in sICAS
**CELL ADHESION MOLECULES**	ICAM-1	Serum	AIS, sICAS	↓	Reduced after 6 months; correlates with perfusion
	ICAM-3	Serum	General inflammation	↓	Reduced in RIC-treated periodontitis patients
	P-selectin	Serum	AIS	Variable	Inconsistent clinical results
**MATRIX METALLOPROTEINASES**	MMP-9	Serum	AIS, sICAS	↓ in sICAS	Significant reduction after 6 months in sICAS; variable in AIS
**TRANSCRIPTION FACTORS**	NF-κB	Serum	AIS	↓	Reduced after 8 weeks RIC
	TLR4	Serum	AIS	↓	Reduced after 8 weeks; inversely correlates with collaterals
**HEAT SHOCK PROTEINS**	HSP27 (total)	Serum	AIS	↑	Increased after 4 days RIC
	HSP27 (phosphorylated)	Serum	AIS	↑	Increased after 4 days; associated with better outcomes
	HSP70	Serum	AIS	↑	Upregulated in conditioning response
**ANGIOGENIC FACTORS**	VEGF	Serum	AIS, sICAS	↑	Elevated after RIC in both AIS and sICAS
	bFGF	Serum	AIS, sICAS	Variable	Increased in sICAS; unchanged in AIS
	EGF	Serum	AIS	↑	Correlates with motor recovery
	PDGF	Serum	AIS	↔	No change observed
**NEUROTROPHIC FACTORS**	BDNF	Serum	AIS, sICAS	↑ in chronic	Increased after 6 months in sICAS; no acute change
**VASCULAR MARKERS**	Endothelin-1	Serum	AIS	↓	Reduced after 6 months; correlates with perfusion
	Homocysteine	Serum	cSVD	↓	Reduced after 1 year; linked to WMH improvement
**COAGULATION & PLATELET FUNCTION**	Fibrinogen	Serum	sICAS, cSVD	↓ in sICAS	Reduced in sICAS; no change in cSVD
	TPA	Serum	sICAS	↑	Increased fibrinolytic activity
	PAI-1	Serum	sICAS	↓	Reduced after 30 days RIC
	Platelet aggregation rate	Whole blood	sICAS	↓	Decreased aggregation
	D-dimer	Serum	Lacunar stroke	↔	No significant change
	Fractal dimension (df)	Whole blood	Lacunar stroke	↑	Increased clot density (paradoxical finding)

 https://doi.org/10.3390/neurosci7020040

Biomarker Category	Specific Biomarker	Sample Type	Disease Context	Effect of RIC	Key Clinical Correlation
HAEMORHEOLOGICAL MARKERS	Erythrocyte aggregation	Whole blood	AIS	↓	Reduced aggregation improves microvascular flow
	RBC deformability	Whole blood	AIS	↔	No change with RIC
	Intracellular NO	RBC	AIS	↔	No change with RIC
	Whole-blood nitrite	Whole blood	AIS	↔	No change with RIC
CELL COUNT-DERIVED INDICES	White blood cell count	Whole blood	sICAS	↓	Reduced after 30 days RIC
	PNR (Platelet-to-Neutrophil Ratio)	Whole blood	AIS	N/A	Low PNR (<30.98) predicts better RIC response
LIPID MARKERS	Total cholesterol	Serum	cSVD	↓	Reduced after 1 year RIC
	LDL cholesterol	Serum	cSVD	↓	Reduced after 1 year RIC
	Triglycerides	Serum	cSVD	↓	Reduced after 1 year RIC
	Lipoprotein(a)	Serum	sICAS	↔	No change but levels >17.4 mg/dL predict RIC benefit
CEREBRAL PERFUSION (IMAGING)	CBF	MRI/CT perfusion	AIS, cSVD	↑	Increased at 90 days post-stroke
	MTT	MRI/CT perfusion	AIS	↓	Decreased at 90 days
	CBV	MRI/CT perfusion	AIS	N/A	Measured but not primary endpoint
	Blood flow velocity (MCA, ACA, PCA, vertebral, basilar)	TCD ultrasound	AIS, sICAS	↑	Improved velocities at 6 months across all vessels
	MCA pulsatility index	TCD ultrasound	cSVD	↓	Reduced after 1 year RIC
	Brachial artery pulsatility index	Doppler ultrasound	cSVD	↓	Reduced after 1 year RIC
STRUCTURAL IMAGING	WMH volume	MRI (FLAIR/T2)	cSVD	↓	Significantly reduced after 1 year RIC
ENDOTHELIAL FUNCTION	BA-FMD (%)	Ultrasound	Chronic stroke, lacunar	Variable	Improved in chronic stroke; variable in acute SVD
EPIGENETIC & RNA MARKERS	DNA methylation patterns	Whole blood	aSAH	Altered	Widespread changes in gene expression
	miRNA let-7a	Serum	Stroke (preclinical)	Normalized	Elevated post-stroke; restored by RIC
	miRNA-143	Serum	Stroke (preclinical)	Normalized	Elevated post-stroke; restored by RIC
RETINAL IMAGING	Retinal vessel density	OCTA	Stroke, cSVD	N/A	Reduced in patients; RIC effects not yet studied
EMERGING BIOMARKERS (PRECLINICAL/PROPOSED)	Kallistatin	Serum	Preclinical	↑	Protects against vascular damage in animal models
	SDF-1α	Serum	Preclinical	↑	Promotes regeneration in stroke models
	GLP-1	Serum	Preclinical	N/A	Neuroprotective; increases CBF in animal models
	Neurofilament light (NF-L)	CSF	cSVD	N/A	Proposed for future RIC trials

Abbreviations: AIS = Acute Ischemic Stroke; sICAS = Symptomatic Intracranial Arterial Stenosis; cSVD = Cerebral Small Vessel Disease; aSAH = Aneurysmal Subarachnoid Hemorrhage; MRI = Magnetic Resonance Imaging; CT = Computed Tomography; TCD = Transcranial Doppler; OCTA = Optical Coherence Tomography Angiography; CSF = Cerebrospinal Fluid; RBC = Red Blood Cell; NO = Nitric Oxide; MCA = Middle Cerebral Artery; ACA = Anterior Cerebral Artery; PCA = Posterior Cerebral Artery; WMH = White Matter Hyperintensities; BA-FMD = Brachial Artery Flow-Mediated Dilation; ↑ = Increased; ↓ = Decreased; ↔ = No change; N/A = Not assessed in clinical RIC trials.

https://doi.org/10.3390/neurosci7020040
